# A splice acceptor site mutation in *TaGW2*-*A1* increases thousand grain weight in tetraploid and hexaploid wheat through wider and longer grains

**DOI:** 10.1007/s00122-016-2686-2

**Published:** 2016-02-16

**Authors:** James Simmonds, Peter Scott, Jemima Brinton, Teresa C. Mestre, Max Bush, Alicia del Blanco, Jorge Dubcovsky, Cristobal Uauy

**Affiliations:** John Innes Centre, Norwich Research Park, Norwich, NR4 7UH UK; University of California Davis, One Shields Avenue, Davis, CA 95616 USA; Howard Hughes Medical Institute, Chevy Chase, MD 20815 USA; CEBAS-CSIC, Espinardo, P.O. Box 164, 30100 Murcia, Spain

## Abstract

*****Key message***:**

**Across 13 experiments the*gw2*-*A1*mutant allele shifts grain size distribution consistently across all grains significantly increasing grain weight (6.6** **%), width (2.8** **%) and length (2.1** **%) in tetraploid and hexaploid wheat.**

**Abstract:**

There is an urgent need to identify, understand and incorporate alleles that benefit yield in polyploid wheat. The rice *OsGW2* gene functions as a negative regulator of grain weight and width and is homologous to the wheat *TaGW2* gene. Previously it was shown that transcript levels of the A-genome homoeologue, *TaGW2*-*A1*, are negatively associated with grain width in hexaploid wheat. In this study we screened the tetraploid Kronos TILLING population to identify mutants in *TaGW2*-*A1*. We identified a G to A transition in the splice acceptor site of exon 5 which leads to mis-splicing in *TaGW2*-*A1*. We backcrossed the mutant allele into tetraploid and hexaploid wheat and generated a series of backcross derived isogenic lines which were evaluated in glasshouse and field conditions. Across 13 experiments the *GW2*-*A1* mutant allele significantly increased thousand grain weight (6.6 %), grain width (2.8 %) and grain length (2.1 %) in tetraploid and hexaploid wheat compared to the wild type allele. In hexaploid wheat, this led to an increase in spike yield since no differences were detected for spikelet or grain number between isogenic lines. The increase in grain width and length was consistent across grains of different sizes, suggesting that the effect of the mutation is stable across the ear and within spikelets. Differences in carpel size and weight between alleles were identified as early as 5 days before anthesis, suggesting that TaGW2-A1 acts on maternal tissue before anthesis to restrict seed size. A single nucleotide polymorphism marker was developed to aid the deployment of the mutant allele into breeding programmes.

**Electronic supplementary material:**

The online version of this article (doi:10.1007/s00122-016-2686-2) contains supplementary material, which is available to authorized users.

## Introduction

Wheat provides approximately 20 % of the calories and 25 % of protein consumed by humankind (FAOSTAT 2015) and is an important source of micronutrients. There are two major types of polyploid wheat; hexaploid wheat (2*n* = 6*x* = 42; ~17 Mb; AABBDD genomes) primarily used for bread and biscuit products and tetraploid durum wheat (2*n* = 4*x* = 28; ~12 Mb; AABB genomes) used for pasta. The three genomes (A, B, and D referred to as homoeologues), have a complementary set of collinear genes that share between 96 and 98 % sequence identity across coding regions (IWGSC 2014; Krasileva et al. 2013). Since most wheat genes are present as two or three close homoeologous copies (IWGSC 2014), mutations in individual genes can be masked by functional complementation by homoeologous genes present in the other genomes (Borrill et al. 2015; Lawrence and Pikaard 2003). This gene redundancy has limited the use of forward genetic screens in wheat and limited the detection of phenotypic effects of natural variants.

Despite a growing human population and rising demand for wheat, low rates of genetic gains for wheat yields (Ray et al. 2013) pose a real threat to global food security. Therefore there is an urgent need to identify, understand and incorporate alleles that increase wheat yield potential across multiple environments. This task is complicated by the polygenic regulation of grain yield and the strong effect of the environment. To facilitate its study, overall grain yield can be broken down into its individual components. This study focuses on thousand grain weight (TGW) and its physical parameters (grain length, width, area), which are inherited in a relatively stable manner and show higher heritability values than overall yield (Kuchel et al. 2007).

Significant advances have been made in our understanding of the genes controlling seed size in the model species rice (reviewed in Xing and Zhang 2010) and *Arabidopsis* (reviewed in Li and Li 2015). The characterised genes have been shown to function in G-protein signalling (Huang et al. 2009), phytohormone biosynthesis/signalling (Jiang et al. 2013), or in the ubiquitin–proteasome pathway (Li et al. 2008; Song et al. 2007; Xia et al. 2013). Specifically in rice, genes with large effects on grain size have been identified revealing an independent genetic control of grain length and width in this species (Fan et al. 2006; Weng et al. 2008). This differs from our limited understanding in polyploid wheat where quantitative trait loci (QTL) for grain size and shape have been identified (Breseghello and Sorrells 2007; Maphosa et al. 2014; Rustgi et al. 2013; Simmonds et al. 2014; Sun et al. 2009; Williams and Sorrells 2014; Zhang et al. 2010), but no gene affecting TGW has yet been cloned.

The rice *Grain Width and Weight 2* (*OsGW2*) gene encodes a previously unknown RING-type E3 ubiquitin ligase and functions as a negative regulator of grain width and weight in rice (Song et al. 2007). The wheat genome has three *GW2* homoeologues, with the A genome copy (*TaGW2*-*A1*) being most closely examined to date. Two studies have identified a significant association between a *TaGW2*-*A1* promoter single nucleotide polymorphism (SNP) and TGW and grain width in Chinese germplasm (Su et al. 2011; Zhang et al. 2013). Despite discrepancies in the haplotype analysis, both studies show a negative relationship between *TaGW2*-*A1* transcript levels and grain width. A separate study identified a single base insertion in the last exon of *TaGW2*-*A1* which results in a truncated protein. This allele was associated with increased TGW and grain width in hexaploid wheat compared to the full length wild type allele in a F_2:3_ population (Yang et al. 2012). These results support a conserved role of *GW2* between wheat and rice as a negative regulator of grain size and weight. However, contradictory results have been reported from RNA interference (RNAi) studies of all three *TaGW2* homoeologues in wheat: whereas Bednarek et al. (2012) reported a reduction in grain weight and size by down-regulation of *TaGW2* homoeologues, Hong et al. (2014) found an increase in equivalent transgenic lines.

TILLING (Targeted Induced Local Lesion IN Genomes) is a powerful reverse genetics approach to characterise gene function in plants (McCallum et al. 2000). Both tetraploid and hexaploid TILLING populations are available in wheat (Uauy et al. 2009) and have been used to characterise genes involved in flowering time (Chen and Dubcovsky 2012; Chen et al. 2014), disease resistance (Fu et al. 2009) and end-use quality traits (Botticella et al. 2011; Hazard et al. 2012, 2014; Slade et al. 2012). The possibility of generating single, double and triple mutant combinations allows researchers to define contributions of specific homoeologues which can be informative for the deployment of mutant alleles for breeding.

In this study we screened a tetraploid TILLING population to identify mutants in *TaGW2*-*A1*. We identified a mutation in a splice acceptor site which leads to mis-splicing in exon 5 of *TaGW2*-*A1*. We backcrossed the mutant allele into tetraploid and hexaploid wheat and generated a series of BC-derived lines which were evaluated in both glasshouse and field conditions. The mutant allele consistently increased grain weight and morphometric parameters in all environments and across all grains of the spike compared to the wild type full length *TaGW2*-*A1* allele. The effect was detected as early as 5 days before anthesis in developing carpels suggesting that GW2 acts on maternal tissue before anthesis to restrict seed size in wheat.

## Materials and methods

### TILLING of *TaGW2*-*A1*

We first used the 454 5*x* raw data of hexaploid wheat Chinese Spring (Brenchley et al. 2012) to reassemble the *TaGW2*-*A1* gene region. These assemblies were combined with wheat ESTs to design primers in the introns flanking *TaGW2*-*A1* exons 2 and 6 (primers T1-T6). A touchdown PCR programme was used to amplify genomic DNA of Kronos and PCR products were cloned directly using a pGEM-T Easy kit (Promega, Southampton, UK) following the manufacturers’ instructions. Miniprep DNA of clones were insert-sequenced by The Genome Analysis Centre (TGAC) using M13 primers. Sequence reads were aligned and assigned to the A genome using the sequence of the flow sorted chromosome arm DNA (Vrána et al. 2000) and the IWGSC survey sequence which became publicly available afterwards (IWGSC 2014). Homoeologous SNPs between the A and B genomes were used to design a 6AS-specific forward primer (T12) to amplify a region of 1269-bp including exons 2–6 (amplicon T12-T6; GenBank accession KP749901) (Simmonds et al. 2014). Primers T12 and T6 were used to screen the first three plates of the tetraploid ethyl methane sulphonate (EMS) TILLING population using previously published protocols (Uauy et al. 2009). From the 20 putative mutants, a homozygous G to A transition in the AG splice acceptor site of exon 5 was identified in Kronos line T4-2235 (GenBank accession KP749902).

Three seeds of Kronos T4-2235 were germinated and genomic DNA was extracted from each plant (2235-1, -2, and -3) (Pallotta et al. 2003). The *TaGW2*-*A1* gene was cloned using primers T12 and T6 and GoTaq Mastermix (Promega, Southampton, UK), and 50 μl PCR reactions were performed using a touch-down programme (66–61 °C) for all three plants. PCR products were checked by agarose electrophoresis and the single specific band from each PCR was excised and purified using a gel extraction kit (Qiagen, Manchester, UK). Sequencing was performed at TGAC using BigDye Terminator v3.1 Cycle Sequencing Kit (Life Technologies, Paisley, UK).

### Splicing of *TaGW2*-*A1* transcripts in T4-2235 plants

Leaf tips from T4-2235-1, -2, and -3, and wild type Kronos plants were harvested and total RNA isolated using TRIzol (Life Technologies). cDNAs were made using 3 μg of total RNA in M-MLV Reverse Transcriptase reactions (Life Technologies). 0.5 μl of cDNA was cloned using primers MF1 and MR1 (Table S1), GoTaq Mastermix and a touch-down programme (65–60 °C). PCR products were ligated into a TOPO vector (Life Technologies), heat-shocked into One Shot *E.coli* cells (Life Technologies) and colonies selected on Spectinomycin LB agar media. DNA from surviving colonies was sequenced at TGAC.

Quantitative reverse-transcription PCR (qRT-PCR) was performed on developing grains and flag leaves of a BC_4_ near isogenic line (NIL) of Kronos carrying the G2373A mutation. RNA and cDNA was made as described above. Transcript levels were determined using forward primers which were designed to specifically amplify the −4 bp and −9 bp transcript of *TaGW2*-*A*, and a common reverse primer (Online Resource 1). Primer efficiencies for the −4 bp (1.04) and −9 bp (1.09) assay were determined using the cDNA from the mutant NIL. qRT-PCR reactions were carried out with a LightCycler 480 instrument (Roche Applied Science, UK) using LightCycler 480 SYBR Green I Master Mix (Roche Applied Science, UK) and the following conditions: 5 min at 95 °C; 40 cycles of 15 s at 95 °C, 15 s at 60 °C, 20 s at 72 °C. The specificity of the amplicon was determined by dissociation curve analysis (from 60 to 95 °C). Transcript levels were normalized with actin (Uauy et al. 2006) and linearized values determined using the (1 + Efficiency)^−ΔΔCT^ method (Schmittgen and Livak 2008). Five biological replicates of both flag leaves and developing grains 15 days post anthesis were used in the analysis and the results were averaged across two independent technical replicates.

### KASPar development

A KASP assay targeting the G2373A SNP was developed (K1-K2/K3; Online Resource 1 and 2) and validated in the three T4-2235 plants. Briefly, two allele specific reverse primers were designed for the G2373A SNP incorporating either the G or A polymorphism at the 3′ end (primer K2 for the wildtype (G) and K3 for the mutant (A) allele). The common primer was developed to discriminate against the B genome by utilising a 3 bp indel present in the A genome (primer K1). Allele specific primers were synthesised with FAM and HEX tails and reactions and cycling conditions were as described before (Trick et al. 2012). Fluorescent end-point genotyping was carried out using a Tecan Safire plate reader (Tecan Group AG, Mannerdorf, Switzerland).

### Plant growth and NIL development

TILLING mutant T4-2235 plant 3 was crossed to Kronos and the resulting F_1_ self-pollinated to produce a segregating F_2_ population. NILs were developed through subsequent backcrossing to Kronos using the K1-K2/K3 KASPar marker for selection. At both the BC_2_ and BC_4_ generations heterozygous plants were self-pollinated and homozygous NILs extracted from the resultant BC_2_F_2_ and BC_4_F_2_ plants. A similar backcrossing scheme was followed for the introgression of the mutation into hexaploid wheat, cv. Paragon.

Plants were grown in lit greenhouses with 16 h light (250–300 μmol m^−2^ s^−1^) and 8 h dark at 18 °C and were sown in Petersfield Cereal Mix (Petersfield, Leicester, UK). In the greenhouse, experimental units (a single plant in a 1 L pot) were organized in a complete randomized design (CRD) for both the Kronos and Paragon germplasm. Field experiments were conducted in Norwich, UK (52°37′39.9″N, 1°10′45.9″E) in 2013, 2014, and 2015 and Tulelake, CA, USA (41°57′47.9″N, 121°28′12.7″W) in 2013, and organized as either CRD (2013–2014) or randomized complete block designs (RCBD; 2015). Experimental units in the field consisted of 1 m rows (F_3_) or ~6.6 m^2^ plots (BC_2_ and BC_4_ NILs) depending on the germplasm being studied.

### Grain phenotyping

Average grain morphometric measurements (grain width, length, area) and thousand grain weight were recorded using the MARVIN seed analyser (GTA Sensorik GmbH, Germany). In glasshouse experiments, values for individual plants were calculated from the means of 3 or 4 ears (F_2_ and F_3_ experiments, respectively), or from all available harvested ears (BC NILs). Values from field experiments were based on a single sample of approximately 400 grains from each experimental unit (1 m row or 6.6 m^2^ plot). These average values were used in the statistical analyses below. In addition, raw individual grain data for each BC-derived plant was extracted and all grains within the same genotypic class were grouped to generate box-and-whisker plots. The objective of this last analysis was to show the effect of the *TaGW2*-*A1* gene on grains of different sizes.

### Carpel/grain developmental time course

Paragon BC_2_ NILs grown in Norwich in 2014–2015 were used for the carpel/grain developmental time course. These genotypes included two independent BC_2_ NILs with the G2373A mutation and two sibling NILs with the wild type Paragon allele. In each of four blocks, we tagged 65 ears per genotype at ear emergence (peduncle just visible) on the same day to ensure that subsequent sampling took place at the same developmental stage. Ten spikes per genotype (per block) were sampled at six time-points: heading (−5 days post anthesis), anthesis (0 dpa), 3, 9, 16, and 23 dpa. From these spikes, ten carpels/grains were sub-sampled from the two outer florets (floret positions F1 and F2) of the five spikelets located in the middle of the spike. The carpels/grains were then weighed (fresh weight), morphometric parameters measured on the MARVIN analyser, and then dried to constant dry weight in an oven at 37 °C. In total ~100 carpels/grains were sampled per block for each genotype at each time-point (10 spikes × 10 carpels/grains). For the statistical analysis, however, the carpels/grains and spikes were considered as sub-samples of the experimental unit and hence the average of the individual genotype within the block was used (i.e. the average of 100 carpels/grains).

### Statistical analysis

The Chi-squared test was performed using the CHITEST function in Excel (Microsoft Office, Microsoft) to evaluate the segregation ratios in the F_2_ Kronos glasshouse experiment. To assess the differences between the *GW2*-*A1* and *gw2*-*A1* alleles, the average grain morphometric parameters and TGW of experimental units were analysed using one-way (GH and Field CRD experiments) and two-way (Field RCBD experiments) analysis of variance (ANOVA). For the F_2_ glasshouse experiment, the effect of the *TaGW2*-*A1* gene was partitioned into additive (linear) and dominant (quadratic) effects using orthogonal contrasts. The three F_3_ experiments were jointly analysed in a two-way ANOVA with the *TaGW2*-*A1* allele and the experiment included in the model (Table 1). Similar two-way ANOVAs were conducted for the five tetraploid BC-derived experiments (Table 2) and the four hexaploid experiments (Table 3). For the carpel/grain time course, a two-way ANOVA including genotype and block was used for the analysis. Differences in expression levels between mutant transcripts were assessed by ANOVA using the technical replicates as a block and the transcript, tissue, and transcript*tissue interaction in the model. ANOVAs were performed using GenStat 15th edition (http://www.vsni.co.uk/).

**Table 1:** Effect of G2373A *gw2*-*A1* allele on thousand grain weight (TGW) and grain morphometric parameters in F_2_ and F_3_ populations of tetraploid wheat Kronos

Experiment	Genotype (allele)	*N*	TGW (g)	Area (mm^2^)	Width (mm)	Length (mm)
F_2_	*GW2*-*A1/GW2*-*A1* (Kronos)	18	50.4 ± 1.4	21.7 ± 0.3	3.73 ± 0.04	7.47 ± 0.05
GH	*GW2*-*A1/gw2*-*A1* (heterozygous)	53	54.1 ± 0.8	22.7 ± 0.2	3.83 ± 0.02	7.65 ± 0.03
	*gw2*-*A1/gw2*-*A1* (G2373A)	18^a^	55.8 ± 1.7	23.1 ± 0.4	3.83 ± 0.04	7.73 ± 0.06
			10.6 %*^,b^	6.8 %**	2.5 %	3.4 %**
F_3_						
GH	*GW2*-*A1/GW2*-*A1* (Kronos)	78	29.5 ± 0.6	18.9 ± 0.1	3.22 ± 0.02	7.33 ± 0.02
	*gw2*-*A1/gw2*-*A1* (G2373A)	80	30.6 ± 0.7	19.5 ± 0.2	3.28 ± 0.02	7.43 ± 0.03
			3.8 %	3.2 %**	2.0 %*	1.5 %**
Field-JIC	*GW2*-*A1/GW2*-*A1* (Kronos)	18	36.3 ± 0.8	20.1 ± 0.2	3.42 ± 0.02	7.3 ± 0.06
	*gw2*-*A1/gw2*-*A1* (G2373A)	19	39.3 ± 1.1	21 ± 0.2	3.52 ± 0.03	7.43 ± 0.06
			8.2 %*	4.7 %**	2.7 %*	1.8 %
Field-UCD	*GW2*-*A1/GW2*-*A1* (Kronos)	18	55.5 ± 0.7	22.9 ± 0.2	3.72 ± 0.02	7.73 ± 0.04
	*gw2*-*A1/gw2*-*A1* (G2373A)	19	57.4 ± 0.6	23.7 ± 0.2	3.74 ± 0.02	7.94 ± 0.03
			3.5 %	3.5 %**	0.6 %	2.7 %***
Overall ANOVA F_3_ populations (*P* value)			0.023	<0.001	0.002	<0.001

Four experiments were conducted across glasshouse (GH) and field environments. *N* corresponds to number of plants (GH) or 1 m rows (field). The overall ANOVA was conducted for the three F_3_ experiments

* (*P* < 0.05), ** (*P* < 0.01), *** (*P* < 0.001)

^a^ In the F_2_ experiment, phenotypic data from a single homozygous mutant line was ambiguously labelled and therefore excluded. This line was recovered in the F_3_ experiments and confirmed through the KASP marker

^b^ The percentages correspond to the difference between the *gw2*-*A1* and *GW2*-*A1* phenotypes as a percentage of the *GW2*-*A1* wild type Kronos allele

**Table 2:** Effect of G2373A *gw2*-*A1* allele on thousand grain weight (TGW) and grain morphometric parameters in tetraploid BC_2_ and BC_4_ lines

Experiment	Genotype (allele)	*N*	TGW (g)	Area (mm^2^)	Width (mm)	Length (mm)
BC_2_F_2_ (GH)	*GW2*-*A1/GW2*-*A1* (Kronos)	15	51.4 ± 0.8	23.6 ± 0.2	3.91 ± 0.02	7.79 ± 0.03
	*gw2*-*A1/gw2*-*A1* (G2373A)	10	55.2 ± 1.3	24.8 ± 0.2	4.06 ± 0.03	7.93 ± 0.04
			7.5 %**^,a^	5.2 %**	3.8 %**	1.8 %**
BC_2_F_3_ (GH)	*GW2*-*A1/GW2*-*A1* (Kronos)	121	50.5 ± 0.4	22.2 ± 0.1	3.95 ± 0.01	7.21 ± 0.02
	*gw2*-*A1/gw2*-*A1* (G2373A)	110	53.2 ± 0.4	23.2 ± 0.1	4.08 ± 0.01	7.32 ± 0.02
			5.4 %***	4.4 %***	3.2 %***	1.6 %***
BC_4_F_2_ (GH)	*GW2*-*A1/GW2*-*A1* (Kronos)	18	72.3 ± 1.3	27.5 ± 0.3	4.28 ± 0.03	8.32 ± 0.05
	*gw2*-*A1/gw2*-*A1* (G2373A)	16	75.5 ± 1.2	29.2 ± 0.3	4.42 ± 0.03	8.57 ± 0.05
			4.4 %	6.1 % **	3.1 % *	3.0 % **
BC_2_F_4_ (Field-14)	*GW2*-*A1/GW2*-*A1* (Kronos)	11	51.3 ± 0.9	23.0 ± 0.2	3.75 ± 0.02	7.76 ± 0.03
	*gw2*-*A1/gw2*-*A1* (G2373A)	8	54.9 ± 0.8	24.2 ± 0.3	3.88 ± 0.03	7.96 ± 0.06
			7.0 %**	5.5 %***	3.5 %**	2.6 %**
BC_2_F_5_ (Field-15)	*GW2*-*A1/GW2*-*A1* (Kronos)	15	58.7 ± 1.0	22.9 ± 0.2	3.60 ± 0.03	8.32 ± 0.03
	*gw2*-*A1/gw2*-*A1* (G2373A)	18	62.5 ± 0.8	24.1 ± 0.3	3.73 ± 0.02	8.47 ± 0.03
			6.4 %**	5.5 %**	3.7 %**	1.7 %**
Overall ANOVA BC populations (*P* value)			<0.001	<0.001	<0.001	<0.001

Experiments were conducted across glasshouse (GH) and field environments (2014 and 2015). N corresponds to number of plants (GH) or plots (field)

* (*P* < 0.05), ** (*P* < 0.01), *** (*P* < 0.001)

^a^ The percentages correspond to the difference between the *gw2*-*A1* and *GW2*-*A1* phenotypes as a percentage of the *GW2*-*A1* wild type Kronos allele

**Table 3:** Effect of G2373A *gw2*-*A1* allele on thousand grain weight (TGW) and grain morphometric parameters in hexaploid BC_2_ Paragon lines

Experiment	Genotype (allele)	*N*	TGW (g)	Area (mm^2^)	Width (mm)	Length (mm)
BC_2_F_3_ (GH)	*GW2*-*A1/GW2*-*A1* (Paragon)	65	37.0 ± 0.3	18.8 ± 0.1	3.74 ± 0.01	6.16 ± 0.01
	*gw2*-*A1/gw2*-*A1* (G2373A)	58	39.7 ± 0.4	19.5 ± 0.1	3.84 ± 0.01	6.26 ± 0.03
			7.1 %***^,a^	4.1 %***	2.6 %***	1.6 %***
BC_2_F_4_ (Field-14)	*GW2*-*A1/GW2*-*A1* (Paragon)	10	43.8 ± 0.3	20.5 ± 0.1	3.76 ± 0.01	6.63 ± 0.01
	*gw2*-*A1/gw2*-*A1* (G2373A)	8	46.9 ± 0.3	21.4 ± 0.1	3.89 ± 0.01	6.73 ± 0.03
			7.0 %***	4.1 %***	3.4 %***	1.6 %**
BC_2_F_5_ (Field-15)	*GW2*-*A1/GW2*-*A1* (Paragon)	10	44.5 ± 0.8	17.9 ± 0.2	3.4 ± 0.02	6.45 ± 0.03
	*gw2*-*A1/gw2*-*A1* (G2373A)	10	47.0 ± 0.8	18.5 ± 0.1	3.48 ± 0.02	6.53 ± 0.03
			5.6 %***	3.3 %***	2.4 %***	1.3 %
BC_4_F_3_ (Field-15)	*GW2*-*A1/GW2*-*A1* (Paragon)	44	47.4 ± 0.3	19.1 ± 0.1	3.62 ± 0.01	6.52 ± 0.01
	*gw2*-*A1/gw2*-*A1* (G2373A)	40	51.9 ± 0.5	20.3 ± 0.1	3.73 ± 0.01	6.69 ± 0.03
			9.4 %***	5.9 %***	3.0 %***	2.7 %***
Overall ANOVA BC populations (*P* value)			<0.001	<0.001	<0.001	<0.001

Experiments were conducted in glasshouse (GH) and field environments (2014 and 2015). N corresponds to number of plants (GH) or plots (field)

** (*P* < 0.01), *** (*P* < 0.001)

^a^ The percentages correspond to the difference between the *gw2*-*A1* and *GW2*-*A1* phenotypes as a percentage of the *GW2*-*A1* wild type Paragon allele

## Results

### Identification and characterisation of the *TaGW2*-*A1* mutant

**Fig. 1:**
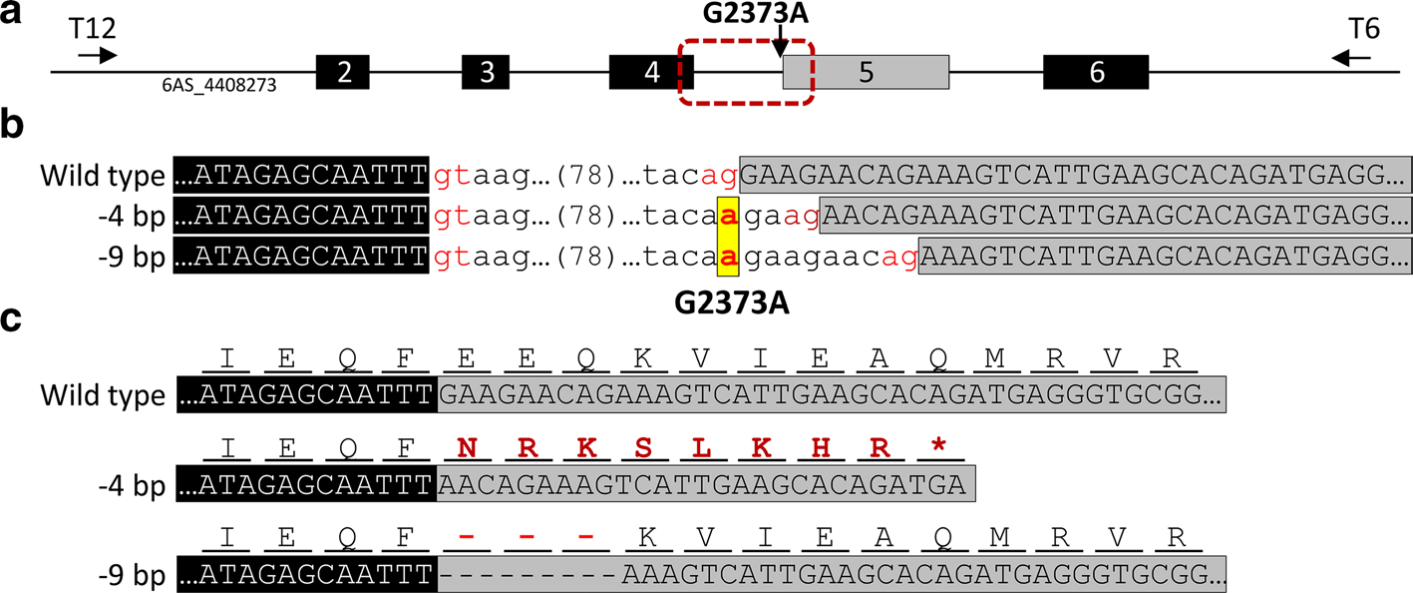
G2373A results in mis-splicing of *TaGW2*-*A1*. **a** Diagram of *TaGW2*-*A1* target region including exons 2–6 (*black and grey numbered boxes*) and introns (*thin line*). The position of the G>A transition at position 2373 (G2373A) of IWGSC_CSS_6AS_scaff_4408273 is indicated. **b** Sequence alignment of gDNA from wild type (*top*) and mutant line T4-2235 (*middle and bottom*) which includes the G2373A transition. The G2373A mutation in line T4-2235 is in *red font* and *yellow highlight*. Exon sequences are in uppercase letters (exon 4 in *black*; exon 5 in *grey*), whereas intron sequences adjacent to the splice sites (*red font*) are in lowercase. The 78-bp of intron four are represented by the *number 78* between parentheses. Note that the mutation leads to a change in the AG splice acceptor site in T4-2235 which removes either four bp (GAAG) or nine bp (GAAGAACAG) from exon 5. **c** Nucleotide sequence of cDNA spanning exons 4 and 5 in wild type (*top sequence*) and the two variants of mutant T4-2235 (*middle and bottom sequences*), with their corresponding amino acid translations. The −4 bp mutant allele is missing four nucleotides which disrupts the reading frame (*red amino acid residues*) leading to a premature termination codon (*red asterisk*) in the T4-2235 sequence. The −9 bp mutant allele is missing nine nucleotides which leads to the loss of three amino acids (EEQ) from the mutant protein

Primers T12 and T6 were used to screen a region of 1269-bp including exons 2–6 in 1152 individuals of the Kronos EMS TILLING population. Among the 20 putative mutants identified within this region, we found a homozygous mutation in the guanine residue of the AG canonical splice acceptor site of exon 5 in Kronos TILLING line T4-2235 (Fig. 1a). Using the recently published wheat chromosome arm survey sequence as a reference (scaffold 6AS_4408273), the G–A transition is 2373-bp from the predicted start codon of *TaGW2*-*A1* and was labelled as G2373A.

The homozygous mutation was confirmed in three independent M_3_ plants derived from T4-2235, all showing the G2373A transition. No additional mutations were found across the *TaGW2*-*A1* coding sequence in T4-2235 plants. A genome-specific KASP assay was developed to facilitate the selection of the mutant G2373A allele during subsequent population development and in marker assisted selection (K1-K2/K3; Online Resource 2).

Several potential alternative AG splice acceptor sites were identified in exon 5 immediately following the EMS mutation. Therefore, we sequenced cDNA from leaves of T4-2235 plants to determine the effect of the G2373A splice site mutation on the *TaGW2*-*A1* transcript. Based on the sequencing of 12 independent clones we found that in nine clones the splicing happened at the AG located 4-bp downstream of the wild type splice site, whereas for three clones splicing occurred after the AG located 9-bp from the wild type site. The −4 bp transcript (Fig. 1b) results in a frame-shift that generates a premature termination codon 27-bp within exon 5 (Fig. 1c). This results in the truncation of the C-terminus 290 amino acids and a shorter protein of only 134 amino acids. The −9 bp transcript results in a shorter, but in-frame, cDNA encoding a GW2 protein missing three amino acids (E127, E128, Q129). We further characterised the relative expression of the −4 and −9 bp transcripts through qRT-PCR of BC_4_ near-isogenic lines of the G2373A allele in the Kronos background. In both grain and leaf tissue we found 15–20-fold higher expression of the −4 bp transcript, compared to the −9 bp transcript (*P* = 0.019). No significant effect of tissue type or tissue*transcript interaction was detected. Thus, based on the cloning and expression data, the mis-splicing of exon 5 from the G2373A mutation results in two types of mutant protein; predominantly a 134 amino acid truncated GW2 protein, and less frequently a GW2 protein missing three amino acids.

### The G2373A mutation leads to increased grain size in tetraploid wheat

**Fig. 2:**
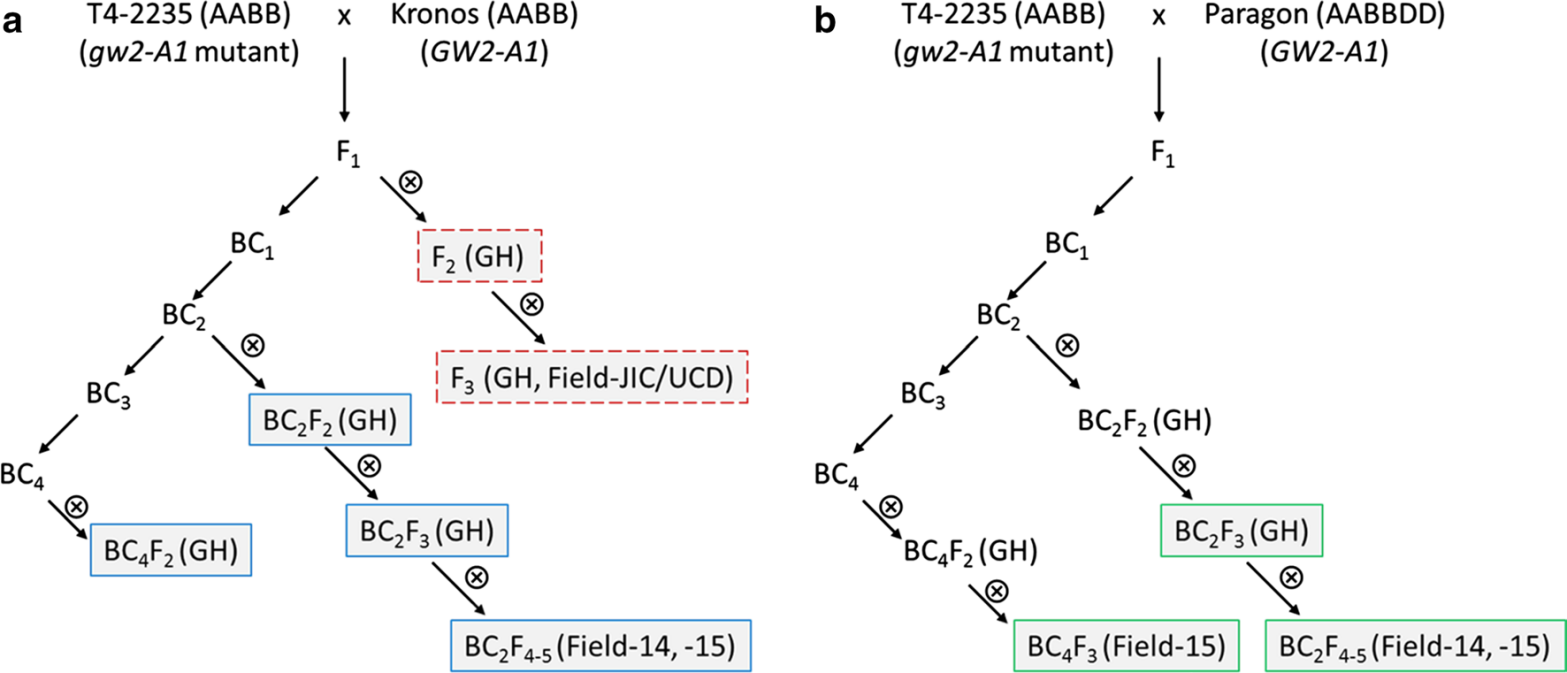
Crossing scheme used to introduce the G2373A allele (*gw2*-*A*) into tetraploid wheat Kronos **a** and hexaploid wheat Paragon **b** both carrying a wild type *GW2*-*A* allele. *Small crossed circles* indicate self-pollination, whereas *straight arrows* indicate back-cross to either Kronos (**a**) or Paragon (**b**). For the Kronos stream (**a**), F_2_-, BC_2_-, and BC_4_-derived lines were evaluated under glasshouse (GH) and field environments, whereas for the Paragon stream (**b**) only BC_2_- and BC_4_-derived lines were evaluated. The results of each generation are indicated by the *outline colour* (*red dash*: Table 1; *blue*: Table 2; Fig. 4a; *green* Tables 3, 4; Fig. 4b)

To assess the effect of the G2373A mutation on grain morphometric parameters we crossed T4-2235 (plant 3) to wild type Kronos to generate segregating populations (Fig. 2a). We first evaluated an F_2_ population of 90 individuals which were genotyped with the K1-K2/K3 KASP assay. We identified 18 homozygous plants for the Kronos wild type, 19 homozygous plants with the G2373A mutant allele, and 53 heterozygous individuals, consistent with the expected 1:2:1 segregation ratio (*χ*^2^ = 2.86; *P* = 0.239). Homozygous plants with the G2373A mutant allele had significantly higher TGW (10.6 %, *P* = 0.04) and larger grain area (6.8 %, *P* = 0.006) than lines with the Kronos wild type allele (Table 1). This was due to a combined effect of wider and longer grains, although only grain length was statistically significant (3.4 %, *P* < 0.01). The effect of the G2373A mutation was additive for TGW, grain area and length as indicated by the highly significant linear contrasts (*P* ≤ 0.01) and the non-significant quadratic contrasts (*P* > 0.27) for each of these traits.

To further assess this effect of G2373A on grain morphometric parameters, the homozygous F_2_ plants for Kronos and G2373A were self-pollinated and grown in three separate experiments. These included a replicated glasshouse experiment (F_3_-GH) and two field trials conducted in Norwich, UK (JIC) and Tulelake, CA (UCD). Table 1 describes the statistical analyses for the experiments separately and combined and for the varying contributions of grain length and width. Across all three experiments, F_3_ plants with the G2373A mutant allele had significantly larger TGW than wild type plants (ranging from 3.5 to 8.2 %, *P* = 0.023), although within experiments this was only significant in the JIC field trials. Grain length and width were also significantly higher in mutant lines (*P* < 0.001 and *P* = 0.002, respectively), although under field conditions only one of these effects was significant in UCD (length) and JIC (width). Across experiments G2373A mutant plants had significantly larger grain area than wild type plants, ranging from 3.2 to 4.7 % (*P* < 0.001). These experiments on F_3_ plants provide further evidence of the significant effect of *TaGW2*-*A1* on grain size parameters in tetraploid wheat, although the relative contributions to grain length and width vary across growing environments.

In addition to the G2373A mutation in *TaGW2*-*A1*, line T4-2235-3 is expected to carry over 2000 mutations in protein coding genes across its genome (Uauy et al. 2009). Therefore, we backcrossed the mutant allele into Kronos to reduce the mutation load and to evaluate the effect of this mutation in a more homogenous genetic background. We evaluated homozygous lines derived from this backcrossing scheme at the BC_2_ and BC_4_ stages in both GH (BC_2-4_) and field (BC_2_; 2014–2015) conditions (Fig. 2a). We identified consistent and highly significant effects of the G2373A mutation on TGW across all five experiments (ranging from 4.4 to 7.5 %; *P* < 0.001) (Table 2). This was due to significant effects on both grain width and length which increased on average 3.5 % (*P* < 0.001) and 2.2 % (*P* < 0.001), respectively, in lines carrying the G2373A *gw2*-*A1* mutant allele. These combined effects led to an overall significant effect on grain area across experiments of 5.4 % (*P* < 0.001). Importantly, the effects on TGW and grain size were consistent across both glasshouse and field environments.

### The G2373A mutation increases TGW in hexaploid wheat

**Fig. 3:**
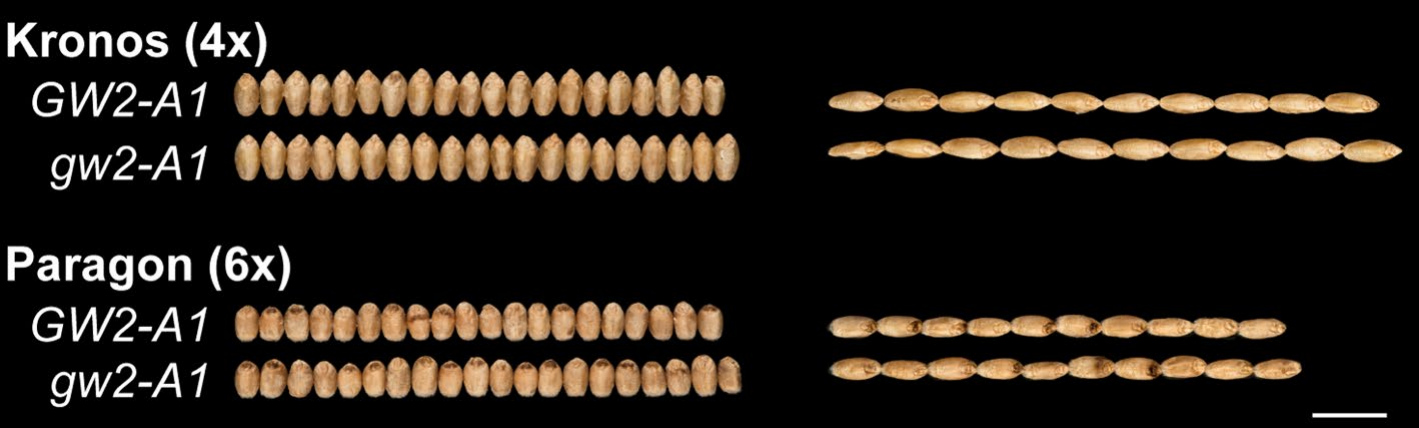
Representative grains from field grown BC_2_F_4_ near isogenic lines carrying the wild type *GW2*-*A1* or G2373A *gw2*-*A1* mutant allele in tetraploid wheat Kronos and hexaploid wheat Paragon. Grains are aligned to show differences in width (20 grains) and length (10 grains). *Scale bar* = 1 cm

To assess the effect of the G2373A mutation in hexaploid wheat we crossed the tetraploid T4-2235-3 mutant plant with hexaploid wheat cultivar Paragon (Fig. 2b). Pentaploid F_1_ plants were crossed again to Paragon and heterozygous BC_1_ individuals were selected using the K1-K2/K3 KASP assay and further backcrossed to Paragon. Experiments were conducted in glasshouse and field environments using homozygous BC_2_ and BC_4_ plants. Across the four environments, the G2373A mutation significantly increased all grain morphometric parameters studied compared to the wild type Paragon allele. The G2373A mutation increased grain area (4.3 %; *P* < 0.001), grain width (2.9 %; *P* < 0.001) and length (1.8 %, *P* < 0.001) resulting in a consistent increase in TGW (7.3 %; *P* < 0.001) (Table 3). The *TaGW2*-*A1* G2373A mutation thus has a consistent effect on the mean value for grain size parameters in both tetraploid and hexaploid wheat. Representative grains from field grown BC-derived lines of Kronos and Paragon are represented in Fig. 3.

We also evaluated the effect of the G2373A mutation on spike yield components in the field grown Paragon BC_2_ and BC_4_ lines in 2015. Across the two experiments we found no significant effects of the mutant allele compared to the wild type allele for number of viable spikelets (*P* = 0.43), seeds per spike (*P* = 0.91) nor seeds per spikelet (*P* = 0.96) (Table 4). In both sets of NILs the G2373A allele led to significant increases in TGW (Table 3); when combined to the non-significant effects on spike yield components this translates into an overall significant increase in the individual spike yield (*P* < 0.001). However, despite the positive effect being present in both BC_2_ (2.7 %) and BC_4_ (9.6 %) NILs, it was only significant in the BC_4_ NILs (Table 4).

**Table 4:** Effect of G2373A allele on spike yield components in hexaploid BC_2_ and BC_4_ Paragon lines

	*GW2* allele	Spike yield	Spikelet number	Seeds/spike	Seeds/spikelet
BC_2_F_5_ (Field-15)	*GW2*-*A1/GW2*-*A1* (Paragon)	2.64 ± 0.15	18.3 ± 0.4	59.0 ± 3.1	3.21 ± 0.13
	*gw2*-*A1/gw2*-*A1* (G2373A)	2.71 ± 0.07	18 ± 0.2	57.6 ± 1.1	3.20 ± 0.05
		2.7 %^a^	−1.8 %	−2.5 %	−0.4 %
BC_4_F_3_ (Field-15)	*GW2*-*A1/GW2*-*A1* (Paragon)	3.25 ± 0.04	19.2 ± 0.1	68.5 ± 0.8	3.32 ± 0.03
	*gw2*-*A1/gw2*-*A1* (G2373A)	3.56 ± 0.09	19.5 ± 0.1	68.7 ± 1.5	3.32 ± 0.06
		9.6 %***	1.2 %*	0.3 %	0.2 %
Overall ANOVA BC populations (*P* value)		<0.001	0.428	0.913	0.959

Values are calculated from the same plants presented in Table 3

* (*P* < 0.05), *** (*P* < 0.001)

^a^ The percentages correspond to the difference between the *gw2*-*A1* and *GW2*-*A1* phenotypes as a percentage of the *GW2*-*A1* wild type Paragon allele

### Distribution of grain morphometric parameters

**Fig. 4:**
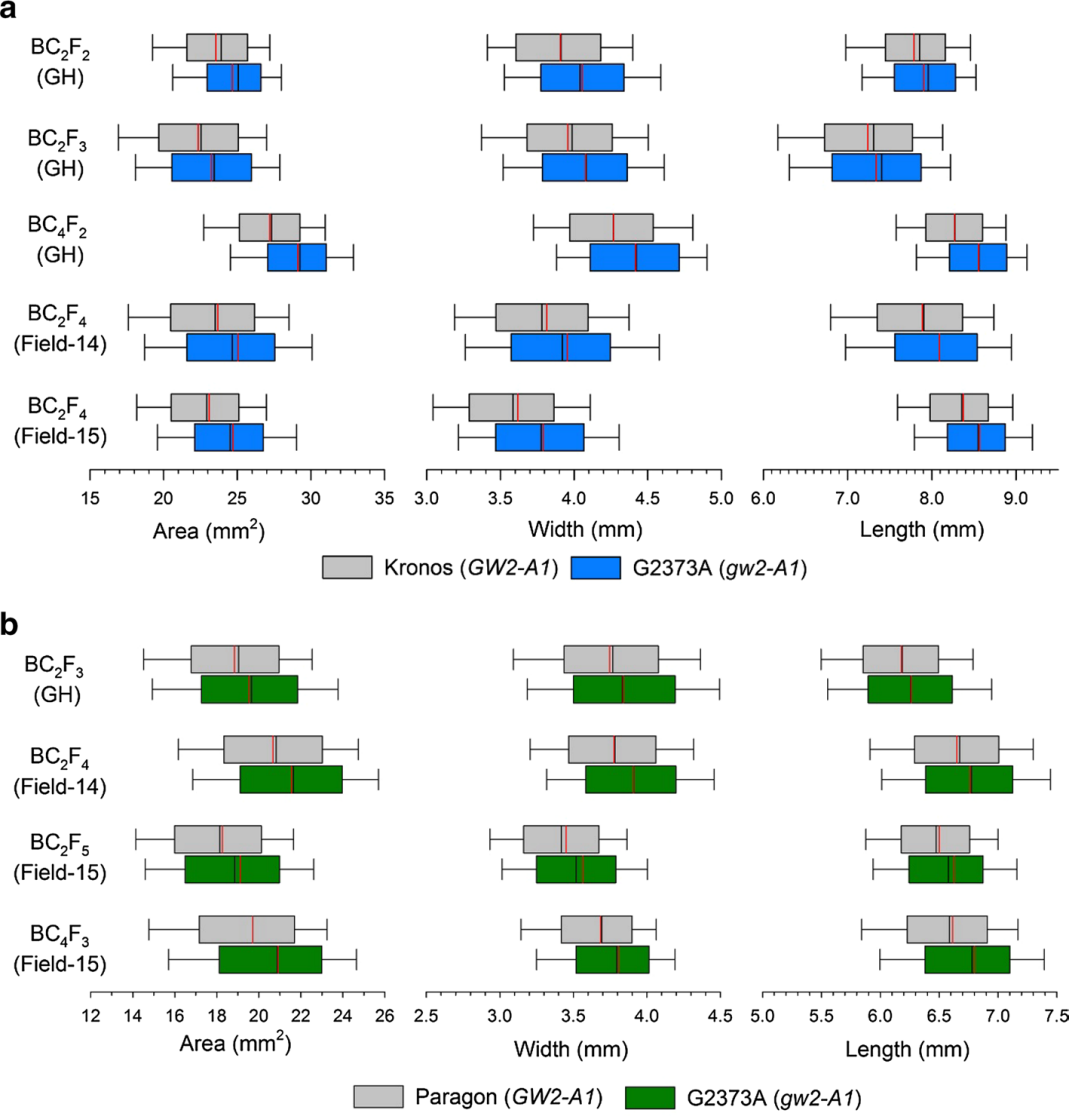
*Box and whisker plots* of grain morphometric parameters of BC-derived lines in tetraploid wheat Kronos **a** and hexaploid wheat Paragon **b**, with the wild type *GW2*-*A* (*grey*) or G2373A *gw2*-*A* mutant allele (*blue* and *green* in tetraploid and hexaploid, respectively). Each *row* represents a separate experiment and the *columns* represents grain *area* (*left*), grain *width* (*middle*) and grain *length* (*right*). The *left* boundary of the *box* indicates the 25th percentile, the *black line* within the *box* marks the median (50th percentile), and the *right* boundary of the *box* indicates the 75th percentile. The *error bars* (*whiskers*) on either side of the *box* indicate the 10th and 90th percentiles. The *red line* within the *box* marks the mean

We further characterised the effect of the mutation by examining the distribution of grains across the different morphometric parameters. The individual values of all measured grains from BC-derived lines were used to generate box and whisker plots (Fig. 4). Across experiments, the G2373A mutation led to an increase in the median and the 5th, 10th, 25th, 75th, 90th, and 95th percentile values (Online Resource 3), in addition to the mean (Tables 2, 3). The mutation thus consistently increases the values of grains across different size categories, suggesting that the effect of the mutation is stable across the ear and within spikelets. This is reflected in the distribution shift for area, width and length in both tetraploid and hexaploid wheat across all experiments (Fig. 4).

### The G2373A mutation increases wheat carpel size

**Fig. 5:**
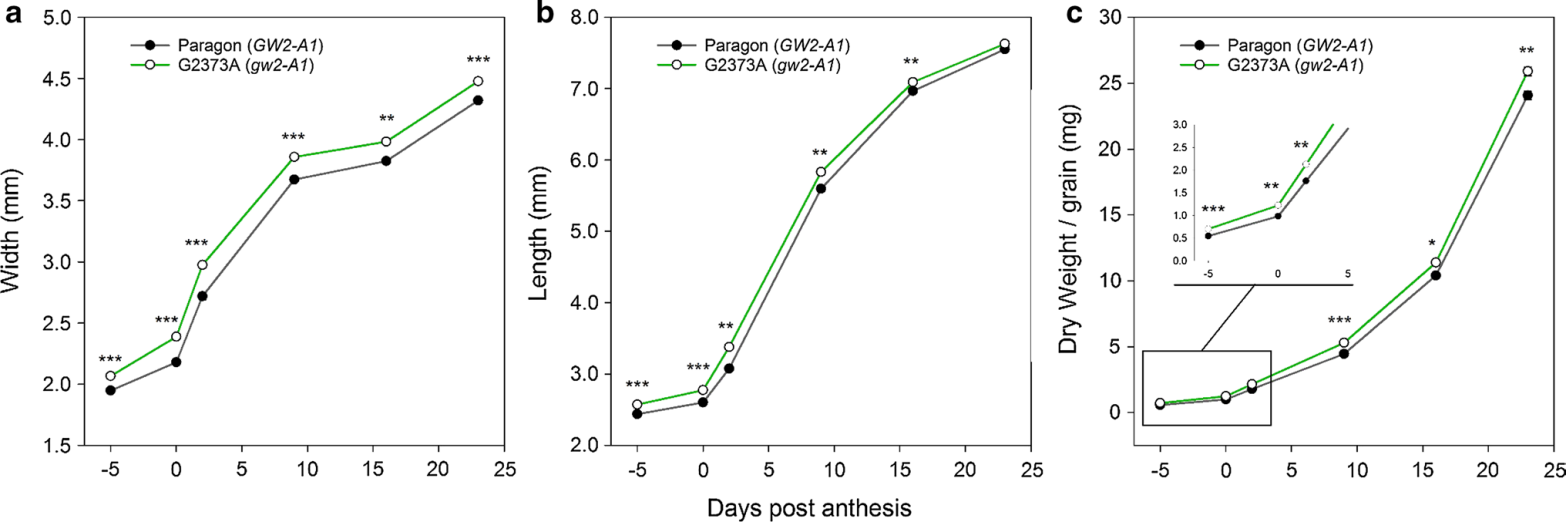
Carpel/grain developmental time course of G2373A *gw2*-*A1* NILs. Carpel/grain width (**a**), length (**b**), and dry weight (**c**) in Paragon BC_2_ NILs carrying either the Paragon wild type *GW2*-*A* (*dark grey*) or the G2373A *gw2*-*A1* mutant allele (*green*). Samples were taken at heading [−5 days post anthesis (dpa)], anthesis (0 dpa) and 3, 9, 16, and 23 dpa. Inset in (**c**) magnifies the first three time points to help visualise differences. *Asterisks* indicate significance of pairwise comparisons at each time point: *P < 0.05; **P < 0.01; ***P < 0.001

We performed a developmental time course of carpel/grain size to assess when the differences in grain morphometric parameters were established. We sampled carpels/grains from field-grown BC_2_ Paragon NILs differing for the G2373A mutation across six time points: heading (−5 dpa), anthesis (0 dpa), 3, 9, 16, and 23 dpa. We identified significant differences from carpel width (6 %) and length (6 %) at heading (Fig. 5a, b; *P* < 0.001). These differences were maintained across the time course for width, whereas for length the differences became less significant after 3 dpa and were non-significant by the middle of grain fill (23 dpa).The increase in carpel/grain size also translated into increased grain dry weight; G2373A BC_2_ NILs had significantly heavier carpels and grains from heading (−5 dpa) onwards (Fig. 5c). Grain filling rates were generally higher in the G2373A NIL compared to the Paragon NIL, although these differences were only significant between 2 and 9 dpa (0.45 vs 0.38 mg/day/grain, respectively; *P* < 0.01). Carpel/grain moisture content between NILs differed by less than 1 % at each time-point (*P* > 0.05) suggesting that the effect of the G2373A mutation was not due to developmental differences but rather to an overall increase in size and weight. These results are consistent with the final harvest values of the same experimental units (BC_2_ 2015; Table 3).

## Discussion

### Is *TaGW2*-*A1* a negative or positive regulator of grain size?

In this study, we show that a splice acceptor site mutation in *TaGW2*-*A1* leads to an increase in TGW in tetraploid and hexaploid wheat. Our results are consistent with the rice *GW2* gene which acts as a negative regulator of grain weight and size (Song et al. 2007). These results are also in agreement with association studies in hexaploid wheat that have identified a negative association between *TaGW2*-*A1* transcript abundance and grain weight and size (mainly width) (Hong et al. 2014; Qin et al. 2014; Song et al. 2007; Su et al. 2011). The negative effect of *TaGW2*-*A1* on grain size has also been described in a traditional bi-parental mapping population (Yang et al. 2012). An allele with a single bp insertion was identified in the last exon of the gene leading to a premature termination codon in hexaploid landrace Lankaodali (328 amino acid peptide). When evaluated in a F_2:3_ mapping population, the mutant *TaGW2*-*A1* allele was associated with significantly increased TGW (10.1 %), grain width (5.8 %) and grain length (2.4 %) across three environments, compared to the wild type (full length) Chinese Spring allele (Yang et al. 2012). Our current study extends these results by showing that a novel *gw2*-*A1* mutant allele increases TGW, grain width and length in tetraploid durum wheat, as well as in hexaploid bread wheat. We developed near isogenic lines to account for background variation associated with the EMS mutagenesis and conducted 13 field and glasshouse experiments which showed that the *gw2*-*A1* mutant allele significantly increases TGW, grain width and length by 6.6, 2.8, and 2.1 %, respectively. A detailed analysis of grain morphometric parameters showed that this effect was consistent across grains of all sizes, suggesting that the effect of the mutation is stable across the ear and within spikelets.

The role of *TaGW2*-*A1* on grain size came into question recently after a study showed that the simultaneous down-regulation of the three *TaGW2* homoeologues by RNAi led to a 30 % reduction in grain weight and an 11 % decrease in grain width in hexaploid wheat (Bednarek et al. 2012). However, an equivalent study reported contrasting results; down-regulation of *TaGW2* homoeologues through RNAi led to an 18 % increase in grain weight and a 5 % increase in grain width in two independent transgenic lines (Hong et al. 2014). Despite the differences between these studies (genetic backgrounds, RNA trigger sequences, growing conditions, etc.) it is difficult to understand the conflicting nature of these results given our current knowledge of *TaGW2*. Different functions of the three *TaGW2* homoeologues, subtle changes in their relative abundance within the transformed genotypes (Hong et al. 2014) and differing levels of down-regulation of the three homoeologues by the RNAi constructs could account for these inconsistencies. It is also possible that GW2 plays additional roles, and that a severe down-regulation of all homoeologues results in a general decrease of fitness that did not allow the plants to capitalize on the potential increase in grain size. We are currently generating *gw2* null mutants combining loss-of-function mutations in all homoeologues to test this hypothesis. Even when considering the discrepant RNAi study, the accumulated association, genetic, mutant and transgenic evidence suggests that *TaGW2*-*A1* functions as a negative regulator of grain weight and grain morphometric parameters in polyploid wheat.

These results suggest a possible conserved role of *GW2* (an E3 ubiquitin ligase) between wheat and rice, and this relationship seems also to extend into other plant species. The overexpression of the rice *GW2* gene in *Arabidopsis* restricts seed (and organ) growth, whereas knockout of the *GW2* homologue (*ArabidopsisDA2* gene) leads to an increase in seed size (Xia et al. 2013). In maize, the *GW2* homologue *ZmGW2*-*CHR4* was shown to be associated with grain weight and width in an association panel and transcript levels were negatively correlated with grain width (Li et al. 2010). Our results on carpel/grain size development suggest that the wheat GW2 protein acts on maternal tissue before anthesis to restrict seed size. This is consistent with the role of *Arabidopsis* homologues DA1 and DA2 which affect cell proliferation in the integument (seed coat) (Li et al. 2008; Xia et al. 2013). Taken together, these results point to a conserved role of *GW2* homologues across plants and support the emerging concept that the ubiquitin pathway contributes to determine final seed size across multiple plant species (Li and Li 2015).

### Phenotypic effects of *GW2* mutants in wheat and rice

The wheat G2373A allele results in the mis-splicing of the *TaGW2*-*A1* transcript leading to two alternative protein forms. The predominant splicing results in a predicted 134 amino acid peptide with a premature truncation of the TaGW2-A1. This is analogous to the rice *gw2* mutant allele which results in a 115 amino acid peptide (Song et al. 2007). The second, less frequent splicing variant results in a protein with a predicted wild type open reading frame, but which lacks three amino acid residues (EEQ). Despite these residues being located 3′ of the conserved RING-domain, they are highly conserved across GW2 homologues in sequenced genomes including monocotyledonous and dicotyledonous species (Online Resource 4). This high degree of conservation suggests a potential functional role for these amino acids and a potential negative effect on GW2 protein function. Additional experiments would be required to test this hypothesis.

The wheat and rice gw2 mutations both affect grain size and shape, however, despite the use of similar germplasm (BC_2_F_2_ populations or more advanced backcross lines) the magnitude of the effect is almost eightfold higher in rice. Near isogenic lines carrying the rice *gw2* mutant allele have 49.8 % higher TGW and 26.2 % wider grains compared to equivalent lines with the wild type *GW2* allele (Song et al. 2007). In tetraploid wheat, BC_2_ and BC_4_ near isogenic lines with the G2373A *gw2*-*A1* allele have 6.2 % higher TGW and 3.5 % wider grains compared to the wild type allele. A similar effect is seen in BC_2_ NILs in hexaploid wheat (7.3 % higher TGW and 2.8 % wider grains in the presence of *gw2*-*A1*).

These results could be simply explained by functional redundancy in polyploid wheat: an *OsGW2* knock-out mutant in rice generates a *gw2* null allele, whereas a *TaGW2*-*A1* mutation only affects the dosage of the gene. The high amino acid conservation between TaGW2 homoeologues (97–98 %) and their similar expression pattern during grain development (Bednarek et al. 2012; Hong et al. 2014), suggests that the *gw2*-*A1* mutant could lead to more subtle phenotypic variation compared to the equivalent rice mutant due to the functional complementation of the B- and D-genomes. The results from the RNAi study of Hong and co-workers (Hong et al. 2014) support this hypothesis since simultaneous down-regulation of all three *TaGW2* homoeologues to ~40 % wild type levels leads to an increase in 18.1 % TGW and 5.4 % wider grains in hexaploid wheat. However, given that these results are from a single experiment with unknown replicate number (Hong et al. 2014) and the contradictory results of Bednarek et al. (2012), caution must be used when interpreting and comparing the absolute values across studies. We cannot rule out that the G2373A splice variant missing three amino acids is able to provide some level of GW2-A1 activity which complements the other genomes. Alternatively, the potential for genetic improvement of grain weight and size through modification of *TaGW2* could be more limited in wheat compared to rice. This could reflect the different morphology of maternal spikelet tissues between wheat and rice (Tashiro and Wardlaw 1989).

The side-by-side evaluation of complete *TaGW2* knockouts (and their factorial combinations) in different genetic backgrounds and environments will help establish putative dosage effects of *TaGW2*. We have identified additional *TaGW2*-*A1* and *TaGW2*-*B1* truncation alleles in the tetraploid Kronos TILLING population and are currently developing the double *TaGW2*-*A1* and *TaGW2*-*B1* null mutant to test these hypotheses. A triple mutant in Paragon is also being developed with a *TaGW2*-*D1* knockout allele. The study of this germplasm will reveal if larger genetic improvements of grain weight and size are achievable through the modification of *TaGW2* beyond those reported in the present study.

### Potential of the G2373A *gw2*-*A1* allele in breeding

The G2373A *gw2*-*A1* allele provides a significant increase in grain weight and size across glasshouse and field conditions in both tetraploid and hexaploid wheat. This increase is consistent across different grain size categories suggesting a stable effect across the length of the spike and within individual spikelets. It will now be important to evaluate if this effect translates into an equivalent increase in yield under field conditions, given the known compensations between yield components in wheat (Slafer et al. 1996). Our results from the BC_2_ and BC_4_ hexaploid NILs show that the mutant allele did not affect spikelet number, seeds per spike and seeds per spikelet in the UK in 2015. This suggests that the grain size effect can translate into overall yield, although additional experiments across multiple locations and genetic backgrounds will be needed to extend these conclusions beyond the genotype and environment tested in this study. From an agronomic point of view, the shift in grain size is also important since it reduces the proportion of small and light grains which can be lost during combine harvesting.

The Kronos and Paragon isogenic lines developed in this study are available to the wheat genetics and breeding communities through the JIC Germplasm Resources Unit (https://seedstor.ac.uk/; accession numbers W10281–W10284) and the US National Small Grains Collection (PI675010-PI675015). We are also currently incorporating the allele into additional European winter wheat varieties and USA and CIMMYT spring wheat and durum lines. This material will help determine the potential of *gw2*-*A1* in improving grain size and yield across environments. The development of the KASPar SNP marker should also help deploy the *gw2*-*A1* allele into additional breeding programmes.

### Use of TILLING mutants to study quantitative variation in polyploids

Understanding the relationship between homoeologues is particularly relevant for improving quantitative traits in wheat, since breeders can utilize allelic variation in each of the homoeologues separately or combined to modulate the desired trait response. The use of stable knockout alleles, such as the TILLING mutant identified in the present study, allows the evaluation of the relative contribution of individual homoeologues and the effects of double and triple mutant combinations (Avni et al. 2014; Botticella et al. 2011; Hazard et al. 2012, 2014; Slade et al. 2012). A relevant example on the use of TILLING mutants for this purpose is provided by work on a grain protein content QTL which was cloned in polyploid wheat (Uauy et al. 2006). The QTL was identified as a frame-shift mutation in the B-genome of a NAC transcription factor (*NAM*-*B1*) which led to a delay in senescence of 2 days and a decrease in protein content of 5–10 %. Simultaneous down-regulation of the different homoeologues by RNAi led to a much stronger phenotype in which senescence was delayed by more than 25 days and protein content was reduced by over 30 %. This broad phenotypic spectrum could be modulated through the use of individual *nam* TILLING mutants (Avni et al. 2014). *nam*-*a1*/*nam*-*d1* double mutants delayed senescence by 20–30 days compared to the wild type plants, whereas in single mutants this delay was between 5 and 10 days. An analogous phenotypic range for grain protein content was achieved through the use of double and single *nam* mutants.

#### Author contribution statement

JS developed the tetraploid and hexaploid backcross populations used in this study, analysed the data and wrote the manuscript; PS led the phenotypic assessments, and provided assistance with field trial preparation and glasshouse husbandry; JB conducted the developmental time course, qRT-PCR analysis and analysed the data; TCM conducted the TILLING screen of *TaGW2*-*A1* and identified the G2373A mutant allele; MB conducted the cDNA sequencing of *gw2*-*A1*; AdB and JD conducted the field trials in Davis; CU conceived the study, analysed the data and wrote the manuscript. All authors read and approved the final manuscript.

## Electronic supplementary material

Below is the link to the electronic supplementary material.

Supplementary material 1 (XLSX 10 kb)

Supplementary material 2 (JPEG 2451 kb)

Supplementary material 3 (XLSX 18 kb)

Supplementary material 4 (PDF 77 kb)
