# Supplementary figures and images for: A splice acceptor site mutation in TaGW2-A1 increases thousand grain weight in tetraploid and hexaploid wheat through wider and longer grains
Source: Theor Appl Genet. 2016 Feb 16;129:1099–112. doi: 10.1007/s00122-016-2686-2 (PMC4869752; doi:10.1007/s00122-016-2686-2)

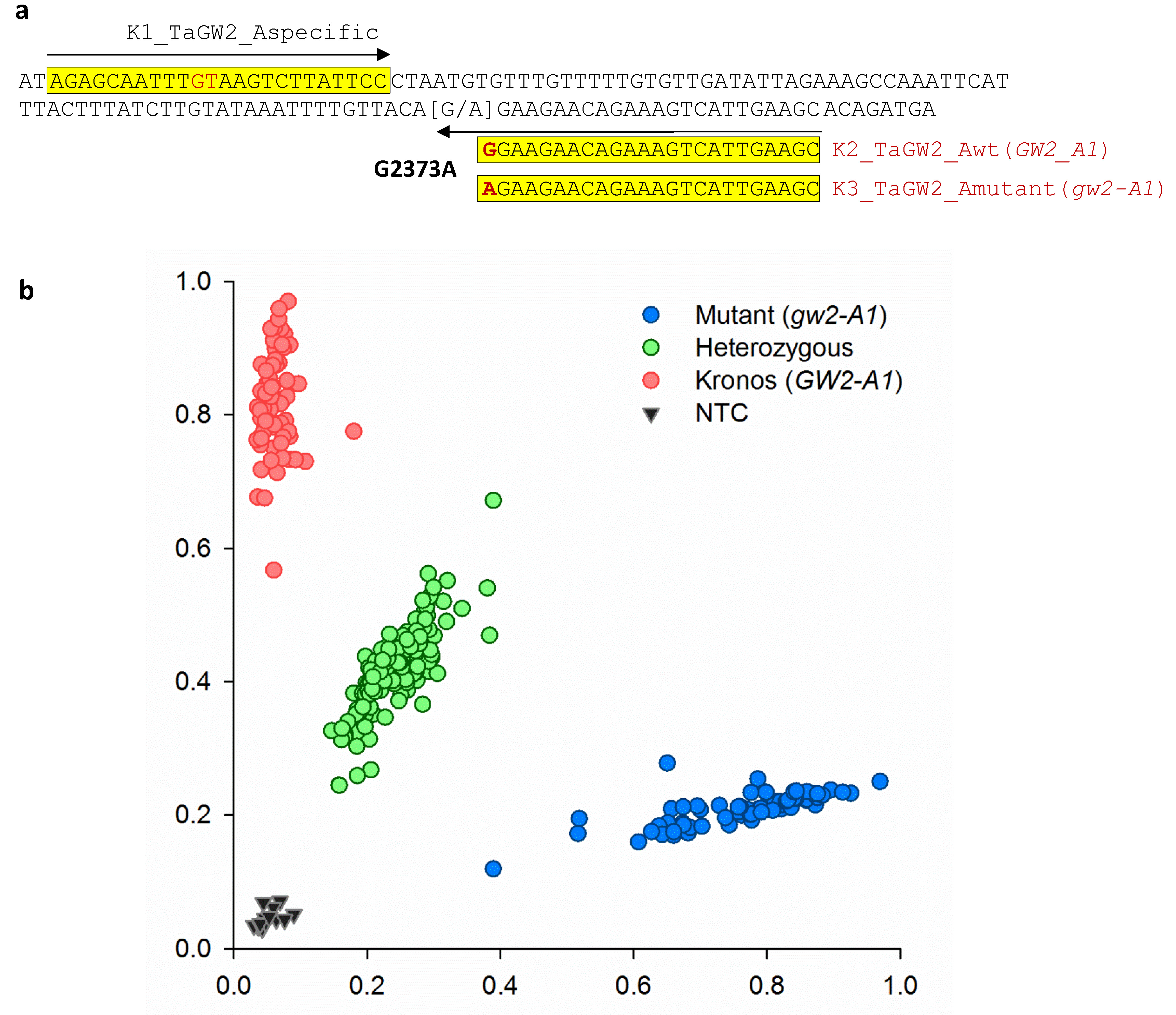

Supplement: Supplementary file 2 — Supplementary material 2 (JPEG 2451 kb) [file 122_2016_2686_MOESM2_ESM.jpg]
