# Supplementary material for: A splice acceptor site mutation in TaGW2-A1 increases thousand grain weight in tetraploid and hexaploid wheat through wider and longer grains
Source: Theor Appl Genet. 2016 Feb 16;129:1099–112. doi: 10.1007/s00122-016-2686-2 (PMC4869752; doi:10.1007/s00122-016-2686-2)
Supplement: Supplementary file 4 — Supplementary material 4 (PDF 77 kb) [file 122_2016_2686_MOESM4_ESM.pdf]

Online Resource 4: Multiple sequence alignment of closely related proteins to TaGW2 using ClustalOmega. In yellow is the conserved RING domain and in grey are the three amino acid residues (EEQ) which are absent from the -9 bp mutant *gw2-A1* transcript. Protein names include GenBank ID followed by the species genus name. The wheat TaGW2-A1 protein is based on the GenBank nucleotide accession number KP749899.

```
TaGW2-A1_Triticum           MGNRIGGRRKAGVEERYTRPQGLYEHRDIDQKKLRKLILEAKLAPCYPGADDAA--GGDLEECPICFLYYPSLNRSKCCSKGICTECFLQMKPTHTARPTQCPFCKTPNYAVEYRGVKTK
ABY51682.1_Hordeum          MGNRIGGRRKAGVEERYTRPQGLYEHRDIDQKKLRKLILETKLAPCYPGADDAA--GADLEECPICFLYYPSLNRSKCCSKGICTECFLQMKPTHTARPTQCPFCKTPNYAVEYRGVKTK
XP_010234090.1_Brachypodium MGNRIGGRRKAGVEERYTRPQGLYEHRDIDQKKLRKLILEAKLAPCYPGADDAA--GGDLEECPICFLYYPSLNRSKCCSKGICTECFLQMKPTHTARPTQCPFCKTPNYAVEYRGVKTK
ABO31101.1_Oryza            MGNRIGGRRKAGVEERYTRPQGLYEHRDIDQKKLRKLILEAKLAPCYMGADDAAA-AADLEECPICFLYYPSLNRSKCCSKGICTECFLQMKPTHTAQPTQCPFCKTPSYAVEYRGVKTK
XP_004951330.1_Setaria      MGNRIGGRRKAGVEERFTRPQGLYEHRDIDQKKLRKLILEAKLAPCYPGADDAPAGGGDLEECPICFLYYPSLNRSKCCSKGICTECFLQMKPTHTARPTQCPFCKTPNYAVEYRGVKTK
XP_002453598.1_Sorghum      MGNRKGGRPKSGGEKRFTPPQGLYEHKDIDQKKLRKLILEAKLAPCYPGADDAAAAGGDLEECPICFLYYPSLNRSKCCSKGICTECFLQMKPTHTARPTQCPFCKTPNYAVEYRGVKTK
XP_008681635.1_Zea          MGNRIGGRRKSGVEERFTRPQGLYDHKDIDQKKLRKLILEAKLAPCYPGADDAAPGGGDLEECPICFLYYPSLNRSKCCSKGICTECFLQMKPTHTARPTQCPFCKTPNYAVEYRGVKTK
XP_009387884.1_Musa         MGNNIRRRR-QMVDEKYTRPQGMYRHREIDYKKLRKLILESKLAPCYPGLEEY---AYDLEECPICFLYYPSLNRSRCCMKGICTECFLQMKPPHSTRPSQCPFCKTSNYAVEYHGAKTK
XP_010925650.1_Elaeis       MGNKIGRRR-QVVDEKYTRPQGLYQHRDIDHKKLRKLILDSKLAPCYPGNEEC---GLDLEECPICFLYYPSLNRSRCCVKGICTECFLQMKPPHSTRPTQCPFCKTPNYAVEYRGMKTK
XP_008801976.1_Phoenix      MGNKIGRRR-QVVDEKYTRPQGLYQHRDIDHKKLRKLILDSKLAPCYPGDDEC---AMDLEECPICFLYYPSLNRSRCCMKGICTECFLQMKAPHSIRPTQCPFCKTSNYAVEYRGMKTK
XP_006302337.1_Capsella     MGNKLGRKR-QVVEERYTKPQGLYVNKDVDIKKLRKLIVESKLAPCYPGDDESC---HDLEECPICFLYYPSLNRSRCCMKSICTECFLQMKNPNSARPTQCPFCKTPNYAVEYRGVKSK
AEE36104.1_Arabidopsis      MGNKLGRKR-QVVEERYTKPQGLYVNKDVDVKKLRKLIVESKLAPCYPGDDESC---HDLEECPICFLYYPSLNRSRCCMKSICTECFLQMKNVNSARPTQCPFCKTPNYAVEYRGVKSK
XP_011653276.1_Cucumis      MGNKLGRRR-QIVDEKYTRPQGLYNHKEVDHKKLRKLILESKLAPCYPGDEESA---SDLEECPICFLYYPSLNRSRCCMKSICTECFLQMKVPNSTRPTQCPYCKTSNYAVEYRGVKSK
XP_004243185.1_Solanum      MGNKLGRRK-QVVDDKYTRPQGLYQHKDVDVKKLRKLILDSKLAPCYPGDDDCP--NANLEECPICFLYYPSLNRSRCCMKGICTECFLQMKTPNSTRPTQCPFCKTSNYAVEYRGVKTK
XP_003543150.1_Glycine      MGNKLGRRR-QVVDEKYTRPQGLYNHKDVDHKKLRKLILESKLAPCYPGDEETA---YDREECPICFLYYPSLNRSRCCTKSICTECFLQMKVPNSTRPTQCPFCKTANYAVEYRGVKSK
XP_010062730.1_Eucalyptus   MGNKLGRRR-QVVDEKYTRPQGLYHHKDVDIKRLRKLILESKLAPCYPGDEEST---CDLEECPICFLYYPSLNRSRCCTKGICTECFLQMKIPNSTRPTPCPFCKTSNYAVEYRGVKTK
XP_008226243.1_Prunus       MGNKLGRRR-QVIDEKYTRPQGLYHHKDVDHKKLKKLILESKLAPCFPGDEECT---CDLEECPICFLYYPSLNRSRCCMKGICTECFLQMKVPNSTRPTQCPFCKTSNYAVEFRGVKSK
CAN66658.1_Vitis            MGNKLGRRR-QVVEDKYTRPQGLYQHKDVDHKKLRKLILDSKLAPCYPGDEEAT---NDFEECPICFLFYPSLNRSRCCTKGICTECFLQMKNPNSTRP-TCPYCKTANYAVEYRGVKTK
XP_007022166.1_Theobroma    MGNKLVRRK-QVVDERYTRPQGLYTHNDVDIKKLRKLILESKLAPCYPGDEECC---CDLEECPICFLYYPSLNRSRCCTKRICTECFLQMKNPNSTRPTQCPFCKTSNYAVEYRGVKTK
                            ***.   :     :.::* ***:* ..::* *:*:***:::*****: * ::      : ********:*******:** * **********  .: :*  **:*** .****::* *:*

TaGW2-A1_Triticum           EERSIEQFEEQKVIEAQMRVRQQALQDEEDKMKRKQSRCSSSRTIAPTTEVEYRDICSTSYSVPSY-QCTQQETECCSSEPSCSAQANMRSFH-----SRHTRDDNIDMNIEDMMVMEAI
ABY51682.1_Hordeum          EERSIEQFEEQKVIEAQMRMRQQALQDEEDKMRRKQSRCSSSRTIAPTTEVEYRDICSTSYSAPPY-RCTEQETECCSSEPSCSAQANMRSFH-----SRHTRDGNIDMNIEDMMVMEAI
XP_010234090.1_Brachypodium EERSIEQLEEQKVIEAQMRMRQQALQDEEDKMKRKQSRCSSSRTIAPTTEVEYRDICSTSYSVPSY-QCTEQEAECCSSEPSCSAQSNMRPVH-----SRHNRDDNIGMNIEEMMVMEAI
ABO31101.1_Oryza            EERSIEQFEEQKVIEAQMRMRQQALQDEEDKMKRKQNRCSSSRTITPTKEVEYRDICSTSFSVPSY-RCAEQETECCSSEPSCSAQTSMRPFH-----SRHNRDDNIDMNIEDMMVMEAI
XP_004951330.1_Setaria      EERSIEQFEEQKVIEAQLRIRQKEIQDEEAKLKRKQSRCSSSRTVTPTKEVEYRDICSTSFSVPSY-QCAEQGTECCSSEPSCSSQASMRPFH-----SRHNRDDNVDMNLEDMMVMEAI
XP_002453598.1_Sorghum      EERSIEQFEEQKVIEAQLRMRQKELQDEEAKMKRKQSRCSSSRTVTPTTEVEYRDICSTSFSVPSY-QCTEQGNECCSSEPSCSSQANMRPFH-----SRHNRDDNVDVNLEDMMVMEAI
XP_008681635.1_Zea          EERSIEQFEEQKVIEAQLRMRQKELQDEEAKMRRKQGRCSSSRTVTPTTEVEYRDICSTSFSVPSY-QCTEQGNECCSSEPSCSSQANRRPFH-----SRHNRDDNVDMNLENMMVMEAI
XP_009387884.1_Musa         EEKGMEQVEEQKVIEAQIRIRQQELQDEAERMKKRQNLSSSSRTMTP-TDVGHHDISNTSTSVPSM-KCSIQSSDLGSRQASCSAPASTRPSQ-----LRQNRDNNLDLDLEDVMVMEAI
XP_010925650.1_Elaeis       EEKGMEQVEEQRVIEAQIRMRQQELQDEAERMKKRQDVSMPSRRMTS-AEVEHRDMCSTSLSVPSF-TCTALGNESVTSQASCSAPASTRPSH-----SRQNRDGNFDLDLEEIMVMEAI
XP_008801976.1_Phoenix      EEKGMEQLEEQRVIEAQIRMRQQEIQDEAERMKKRKDVSSSSGIMTP-AEVEYQDICSTSLS----------GNESVSSQASCSSPDSTRPSH-----SRQNRDDNFDLELEDMMVMEAI
XP_006302337.1_Capsella     EEKGIEQVEEQRVIEAKIRIRQKEMQDDEEKMQKRLESCSSSTSAMT-GEMEYG-----SASAVSY-NSPMDEGETASSQNA---SAVRQHSR-----PRGNRDDEVDVDLEELMVMEAI
AEE36104.1_Arabidopsis      EEKGIEQVEEQRVIEAKIRMRQKEMQDDEEKMQKRLESCSSSTSAMT-GEMEYG-----STSAISY-NSLMDDGEIAPSQNA---SVVRQHSR-----PRGNREDEVDVDLEELMVMEAI
XP_011653276.1_Cucumis      EEKSLEQIEEQRVIEAKIRIRQQELQDDEERMQKRHELSTSNADTTV-E------------NVPSS-QSPAEDEEIVSLQDPCMTQIRPPPPPIPIRSNRNFRDDEFDLDLEDIMVMEAI
XP_004243185.1_Solanum      EEKGIEQIEEQRVIEAKIRMRQQELQDDEEKMHKRRELSTSSSIAGP-SEIEYC-----STAAPSF-ASAVEGGEVVTSQETCTAPTTRQPQR-----TRQNREDDFDLDLEDIMVMEAI
XP_003543150.1_Glycine      EEKGLEQIEEQRVIEAKIRMRQQELQDEEERMHKRLEMSSSNVNVAV-ADVEYS-----SNAVSSSSVSVVENDEIVSSQDSCATSVVRANAT-----TRTNRDDEFDVDLEDIMVMEAI
XP_010062730.1_Eucalyptus   EEKGIEQIEEQRVIEAKIRMRQKELQDEEERMQKRQDLSSSSRTMAP-VEIDNG-----SVGVQSR-RSPVDEEEIVSADQSYSTSISRQPPH-----LRTNRDDEFDLDLEDIMVMEAI
XP_008226243.1_Prunus       EEKGLEQIEEQRVIEAKIRMRQQEIQDEEERMQIRQLLSSSSRNMAP-VGDEYS-----V-TVPSS-ASPAEGEEIVSSQDSCAASMFRQPPP-----PRVYREDEFDLDLEDIMVMEAI
CAN66658.1_Vitis            EEKGMEQIEEQRVIEAKIRMRQKEIQDEEERMQKRQEISSSSSILAQ-GEVEYS-----TTAVPSF-RSPVEGDEIDSSQDPRAASMIIQTLP-----PRQNRDEEFDLDLEDIMVMEAI
XP_007022166.1_Theobroma    EEKGIEQIEEQRVIEAQIRMRQQELQDEEERMQKRQELSSSSTAVAP-GEVQYS-----SVAARS-----SGEEEIVSSQDSQAASMIRQPSH-----PRANRNEEFDIDLEEIMVMEAI
                            **:.:**.***:****::*:**: :**:  ::: :   .  .                                :    :                   *  *: :. :::*::******
```

Simmonds et al. *A splice acceptor site mutation in TaGW2-A1 increases thousand grain weight in tetraploid and hexaploid wheat through wider and longer grains*

```
TaGW2-A1_Triticum           WRSIQEQGSIGNPSCGSFMPF-----EQPT-RERQAFVAAPP----------LEMPHPGGFSCAVAAMAEHQPSSMDFSYM-TGSSAFPVFDMFRRPCNIAGGSMGAAES-SPDSW----
ABY51682.1_Hordeum          WRSIQEQGSIGNPACGSFMPF-----EQPT-RERQAFVAASP----------LEIPHPGGFSCAVAAMTEHQPSSMDFSYM-TGSSAFPVFDMFRRPCNIAGGSLRAVES-SLDSW----
XP_010234090.1_Brachypodium WRSIQEQGSMGNPVCGNFMPV-----IEPPSRERQAF-VPAP----------LEIPHPGGFSCAVASMAEHQPPSMDFSYM-AGNSAFPVFDMFRRQCNISGGSMCAVDS-SPDSW----
ABO31101.1_Oryza            WRSIQEQGSIGNPVCGNFMPV-----TEPSPRERQPFVPAAS----------LEIPHGGGFSCAVAAMAEHQPPSMDFSYM-AGSSAFPVFDMFRRPCNIAGGSMCNLES-SPESW----
XP_004951330.1_Setaria      WRSIQEQGHLVNPVCGSYFPV-----IEPQTRERQAFLPAAP----------MEMPHPGGYSCAVAALAEHQPPSMDFSYM-AGSSTFPVYDMIRRPCNISGGSMCAVENSSLDTW----
XP_002453598.1_Sorghum      WRSIQEQGHLVNPVCGSYFPV-----IEPPSRERQAFLPAAP----------LEMPHPGGYSCAVAALAEHQPASMDFSYM-AGSSTYPVFDMIRRPCNMSSGSLCGVENSSLDTW----
XP_008681635.1_Zea          WRSIQEQGHLVNPVCGSYFPV-----IEPPSRERQAFLPAAP----------LEMPHPGGYSCV-AALAEHQPPSMDFSYM-AGSSTYPVFDMIRRPCNMSSGSLCSVENSSLDTW----
XP_009387884.1_Musa         WLSIQEQGSQGNPSCVGSYLP-----RPSTSGEWHSSHGTAP----------MRPSSSGGLACAAAALAERQHMHGNSAVH-MAIDNAAACDMLQRSGSSSPGITRFVHDNPSGRW----
XP_010925650.1_Elaeis       WLSIQEQGSPGNPPCGGDVLP-----EPSFSD--NSVA--VA----------PVEASPGGLACAVAALAERQHINGDSAAG-MAGSDASSFDMLRQPSSLPVGMATAAENDPPGSW----
XP_008801976.1_Phoenix      WLSIQEQGAPGNPTCGSNVLP-----EPSVEECYNSLA--VP----------PIEVSPGGLACAVASLAEHQHMNGDSAAS-MAGSGALAFDMVRQSSSLPVGMTRVIGKNPPGFW----
XP_006302337.1_Capsella     WLSVQETGTQRNSASGEMTS-----SRQYETE-DHSYVSQPPPMASTVEPAMPSSSSSGGITCAIAALAERQMVGESSN--HNHNVNVSSYSMLPGNCDSYYDIEQEVDDIGNHHHHHQQ
AEE36104.1_Arabidopsis      WLSVQETGTQRNSASGEITS-----SRQYVTD-NHSYVSSPPRVTPIVEPATPSS-SSGGLSCAISALAERQMVGESSSHNHNHNVNVSSYSMLPGNCDSYYDIEQEVDGIDNHHHHRH-
XP_011653276.1_Cucumis      WLSIQEKGRNKDPVYAEAAS-----SDQYATE-GRYVSPATIP------LAGTSSSPSGGLACAIAALAEQQQIGRGPSST-CTNGDSPVFTMLPGATEFYNRMNTNVENYPPTQGSISA
XP_004243185.1_Solanum      WLSIQENGRNRSMDYSDVGP-----SEQYTEE-DHRCVPAVNP-----TASGSSSSPSGGLACAIAALAERQQMSGESSSY--SRNMSSSYNEHPSCDRFSNGEELENDDYPHAEG----
XP_003543150.1_Glycine      WLSIQENGRRRNLSFVDATSGHYVADGRYVSS-VSSVSSVMGP------PTGSSSSPSGGLACAIAALAERQQMAGESSMS-LTNENMPSFNTLPGSRRFYNRLGRDMANYPPGDN----
XP_010062730.1_Eucalyptus   WLSIQEKGKFNSLAQSEAAP-----SEQYVSE-DHYVSQATAP------LAGSGSSPSGGLACAIAALAERQQMSGESSFS--HNTTMSTYNVPPNCGQFYPTAVQDSDGYAAPES----
XP_008226243.1_Prunus       WLSIQENGRHKSPSYGDVTP-----SEQFVTR-QSYVSPAMVS------VSG-SSSPSGGLACAIAALAERQQTSGESSTN--PGGNVPGFSMVPGTSRFYNRVDRESENYSAAVS----
CAN66658.1_Vitis            WLSIQDNGRHRNPLYGDTTT-----AEQYVTE-EHYVLPAMAP------QVESSSSPSGGLACAIAALAERQQMGGESSTN--YNGNMPAFNMPPGSSRFSNRVEQYPENYPPIES----
XP_007022166.1_Theobroma    WQSIQENNRQRNSNYGDAAAS----SVQYVSG-DRYISPAMTT------VAGSSTSPSGGLACAIAALAERQQMSGESSLN--HNGDIPSFNMLCSGSRLYNRVDRVVENYPPAES----
                            * *:*:     .                                              ** :*. ::::*:*

TaGW2-A1_Triticum           ----------SGIAPSCSRREVVREEGECSTDHLSEGAEAGT--SYAGSDIVVDAGTMLPLPFAD----NYSMVASHFRPESIEEQMMYSMAVSLAEA-HGRTH--TQGLAWL-
ABY51682.1_Hordeum          ----------SGIAPSGTRREMVREEGECSIDHWSEGAEAGT--SYAGSDIMADAGTMPPLPFAD----NYSMAASHFRPESIEEQMMYSMAVSLAEA-HGRTH--TQGLTWL-
XP_010234090.1_Brachypodium ----------SGIPPSCSR-EMIREEGECSTDHWSEGAEAGT--SYAGSDIVADAGTMQQLPFAE----NYNMAPSHFRPESIEEQMMYSMTVSLAEA-HGRTH--SQGLAWL-
ABO31101.1_Oryza            ----------SGIAPSCSR-EVVREEGECSADHWSEGAEAGT--SYAGSDIVADAGTMPQLPFAE----NFAMAPSHFRPESIEEQMMFSMALSLADG-HGRTH--SQGLAWL-
XP_004951330.1_Setaria      ----------SGIAPSCSR-EVLREEGECSTDHWSEGAEAGT--SYAGSDIMADAGSMQPLPFAE----NFAMAPSHFRPDSIEEQMMFSMAVSLTDAHHGRAH--AQGMAWL-
XP_002453598.1_Sorghum      ----------SGIAPSCSR-EVVREEGECSTDHWSEGAEAGT--SYAGSDIMADTGTMQPLPFAE----NFTMAPSHFRPESIEEQMMFSMAVSLAEAHHGRTQ--AQGLAWL-
XP_008681635.1_Zea          ----------SGMAPNCSR-GVVREEGECSTDHWSEGAEAGT--SYAGSDIMVDAGAMQPLPFAE----NFTMAPSHFRPESIEEQMMFSMAVSLAEGHHGRTQ--AQGLAWL-
XP_009387884.1_Musa         ----------TEISPDNGREVHGQEFGECLADHQSEVAEAGT--SYNVE-----TGAMT-GPLSE----GVSIPPGHFVPESFEEQMMLAMAVSLAEA-RARMS--NHGVSLL-
XP_010925650.1_Elaeis       ----------NEVLPDSGRGVTR-EEGECSTDHWSEVTEGGT--NYAGSDVRVHARSAA-VSLPE----AGSMAVSHLLPESFEEQMMLAMAVSLADA-GARTN--AQGLTWL-
XP_008801976.1_Phoenix      ----------NEVSPDSGREVPR-EEGECSTDHWSEVTEAGT--SYAGSDVMVDAGAAA-ASFPG----V-NMAANHLLPESFEEQMMLATAVSLAEA-RARTN--AQGLTWL-
XP_006302337.1_Capsella     QHY----------------------------HNNTEMGETGSSNNYVSSYM-----------TGESFHNFPPPPPLVIVPESFEEQMMMAMAVSLAEV-HATTTSASTEVTWQ-
AEE36104.1_Arabidopsis      ----------------------------------HYEMGETGSSNSYVSSYM-----------TGEGFHNFPPPPPLVIVPESFEEQMMMAMAVSMAEV-HATTTCAPTEVTWQ-
XP_011653276.1_Cucumis      ENYAPAQVSTVDTVPDCRM-ILTRNDGEWNLDHQSEEAEAGT--SYPTSDLNEDNSTECALPTVNAMDGGNQ-ATIPIIPQNFEEQMMLAMAVSLAEA-REVST--GPGHSWQE
XP_004243185.1_Solanum      --------T-MHVSPESHL-EIPREDGEWA-DHGSMVAEVGT--SYAVSDEMEDDA---SFPLQGEMVSDLQPNTSSIVPENFEEQMMLAMAVSLAEA-SARSS--PPGVAWH-
XP_003543150.1_Glycine      --------L-NEEPLDEAV-TMTRSHGEWDMDHGTQLTETAT--SYTNSVAAEDRGELSSLPRSDDNDGSLQSATEPIVPESFEEQMMLAMAVSLAEA-RAMSS--GQSASWQ-
XP_010062730.1_Eucalyptus   --------GTTELVHGGGI-PLARDDGEWNMDRGSEVAEAGT--SYASSDSPEDAGGISALPQPNEVDVGFQNIPAPIVPESFEEQMMLAMAVSLAEA-RAVTS--GPGITWQ-
XP_008226243.1_Prunus       --------S-SEMSINHGM-PLTQDDREWNADT-------RT--SYASSDTTEDAGSTSAPPTGNEIEGDLQNVPDPIVPESFEEQMMLAMAVSLAEA-RAVAS--GPGVPWQ-
CAN66658.1_Vitis            --------S-MDALPDGGL-AVTKDDGEWGVDRGSEVAEAGT--SYASSDATDEAGGVAALPPTDEAEGSFQNVGGPIVPESFEEQMMLAMAVSLAEA-RARTS--TQGV-WQ-
XP_007022166.1_Theobroma    --------P-VDMPADGGM-TPARDEGEWGVDHGSEVAEAGT--SYASSDVTEDAGGICTISQQDDIRGSFHNVPGPIVPESYEEQMMLAMAVSLAEA-RAMTS--GPGVSWQ-
                                                                     :  .*  .                            : *:. ***** : ::*:::
```
